# SAGES consensus recommendations on surgical video data use, structure, and exploration (for research in artificial intelligence, clinical quality improvement, and surgical education)

**DOI:** 10.1007/s00464-023-10288-3

**Published:** 2023-07-29

**Authors:** Jennifer A. Eckhoff, Guy Rosman, Maria S. Altieri, Stefanie Speidel, Danail Stoyanov, Mehran Anvari, Lena Meier-Hein, Keno März, Pierre Jannin, Carla Pugh, Martin Wagner, Elan Witkowski, Paresh Shaw, Amin Madani, Yutong Ban, Thomas Ward, Filippo Filicori, Nicolas Padoy, Mark Talamini, Ozanan R. Meireles

**Affiliations:** 1 Surgical Artificial Intelligence and Innovation Laboratory, Department of Surgery, Massachusetts General Hospital, 15 Parkman Street, WAC339, Boston, MA 02114 USA; 2 Department of General, Visceral, Tumor and Transplant Surgery, University Hospital Cologne, Kerpenerstrasse 62, 50937 Cologne, Germany; 3 Computer Science and Artificial Intelligence Laboratory, Massachusetts Institute of Technology, 32 Vassar St, Cambridge, MA 02139 USA; 4 Stony Brook University Hospital, Washington University in St. Louis, 101 Nicolls Rd, Stony Brook, NY 11794 USA; 5 National Center for Tumor Diseases (NCT), Fiedlerstraße 23, 01307 Dresden, Germany; 6 University College London, 43-45 Foley Street, London, W1W 7TY UK; 7 Center for Surgical Invention and Innovation, Department of Surgery, McMaster University, Hamilton, ON Canada; 8 German Cancer Research Center, Deutsches Krebsforschungszentrum (DKFZ), Im Neuenheimer Feld 280, 69120 Heidelberg, Germany; 9 MediCIS, University of Rennes - Campus Beaulieu, 2 Av. du Professeur Léon Bernard, 35043 Rennes, France; 10 Department of Surgery, Stanford School of Medicine, 291 Campus Drive, Stanford, CA 94305 USA; 11 Department of Surgery, University Hospital Heidelberg, Im Neuenheimer Feld 420, 69120 Heidelberg, Germany; 12 New York University Langone, 530 1St Ave. Floor 12, New York, NY 10016 USA; 13 Surgical Artifcial Intelligence Research Academy, Department of Surgery, University Health Network, Toronto, ON Canada; 14 Intraoperative Performance Analytics Laboratory (IPAL), Department of General Surgery, Northwell Health, Lenox Hill Hospital, New York, NY USA; 15 Ihu Strasbourg - Institute Surgery Guided Par L’image, 1 Pl. de L’Hôpital, 67000 Strasbourg, France; 16 Donald and Barbara Zucker School of Medicine at Hofstra/Northwell, Hempstead, NY USA

**Keywords:** AI, Education, Surgical data science, Surgical AI, Delphi consensus

## Abstract

**Background:**

Surgery generates a vast amount of data from each procedure. Particularly video data provides significant value for surgical research, clinical outcome assessment, quality control, and education. The data lifecycle is influenced by various factors, including data structure, acquisition, storage, and sharing; data use and exploration, and finally data governance, which encompasses all ethical and legal regulations associated with the data. There is a universal need among stakeholders in surgical data science to establish standardized frameworks that address all aspects of this lifecycle to ensure data quality and purpose.

**Methods:**

Working groups were formed, among 48 representatives from academia and industry, including clinicians, computer scientists and industry representatives. These working groups focused on: Data Use, Data Structure, Data Exploration, and Data Governance. After working group and panel discussions, a modified Delphi process was conducted.

**Results:**

The resulting Delphi consensus provides conceptualized and structured recommendations for each domain related to surgical video data. We identified the key stakeholders within the data lifecycle and formulated comprehensive, easily understandable, and widely applicable guidelines for data utilization. Standardization of data structure should encompass format and quality, data sources, documentation, metadata, and account for biases within the data. To foster scientific data exploration, datasets should reflect diversity and remain adaptable to future applications. Data governance must be transparent to all stakeholders, addressing legal and ethical considerations surrounding the data.

**Conclusion:**

This consensus presents essential recommendations around the generation of standardized and diverse surgical video databanks, accounting for multiple stakeholders involved in data generation and use throughout its lifecycle. Following the SAGES annotation framework, we lay the foundation for standardization of data use, structure, and exploration. A detailed exploration of requirements for adequate data governance will follow.

The digital revolution and the seemingly widespread access to healthcare data have bestowed the field of medicine with unprecedented opportunities for research, technological innovation, and education. In the late 1980s and early 1990s, laparoscopic surgery began to gain widespread acceptance, its adoption initiated the generation of a new type of data, surgical video. Videos are composed of still images (i.e., frames) played over time and provide both spatial and temporal information, including the nature of interaction between subjects and objects. In a surgical video, the surgeon acts as a subject, altering the operating field, the object, to achieve a specific goal [1]. Today, every second, the collective of all surgical procedures performed worldwide generates vast amounts of surgical video data, along with its associated meta-data. The rapidly growing number of applications of surgical video data result in increasingly complex requirements for data structure and management. Additionally, the exceeding number of use cases for surgical video data leads to a growing need for adequate data governance and the use advanced tools, such as computer vision, machine learning (ML), and artificial intelligence (AI), to allow for enhanced exploration of this type of data.

The data lifecycle (Fig. 1) highlights various interconnected stages that must be considered throughout the evolution of surgical video data—from its creation, storage, and distribution to model creation and utilization, and on to data-driven improvement and future expansions. Guidelines for surgical video data must address the complex aspects around the data in its raw, original state as collected in the operating room, its innate structure, and its associated metadata, which influences all subsequent data management and usage. This is followed by necessary and feasible data manipulation to facilitate sustainable, responsible and practical data storage and exchange. Next, methods for exploring and processing the data must be developed to accommodate current and future use cases and the interests of diverse stakeholders in the broader realm of research, education, and clinical practice. Ultimately, all-encompassing ethical and legal considerations must be factored in to govern data access and address inclusivity, bias, privacy, and consent concerns.

**Fig. 1:**
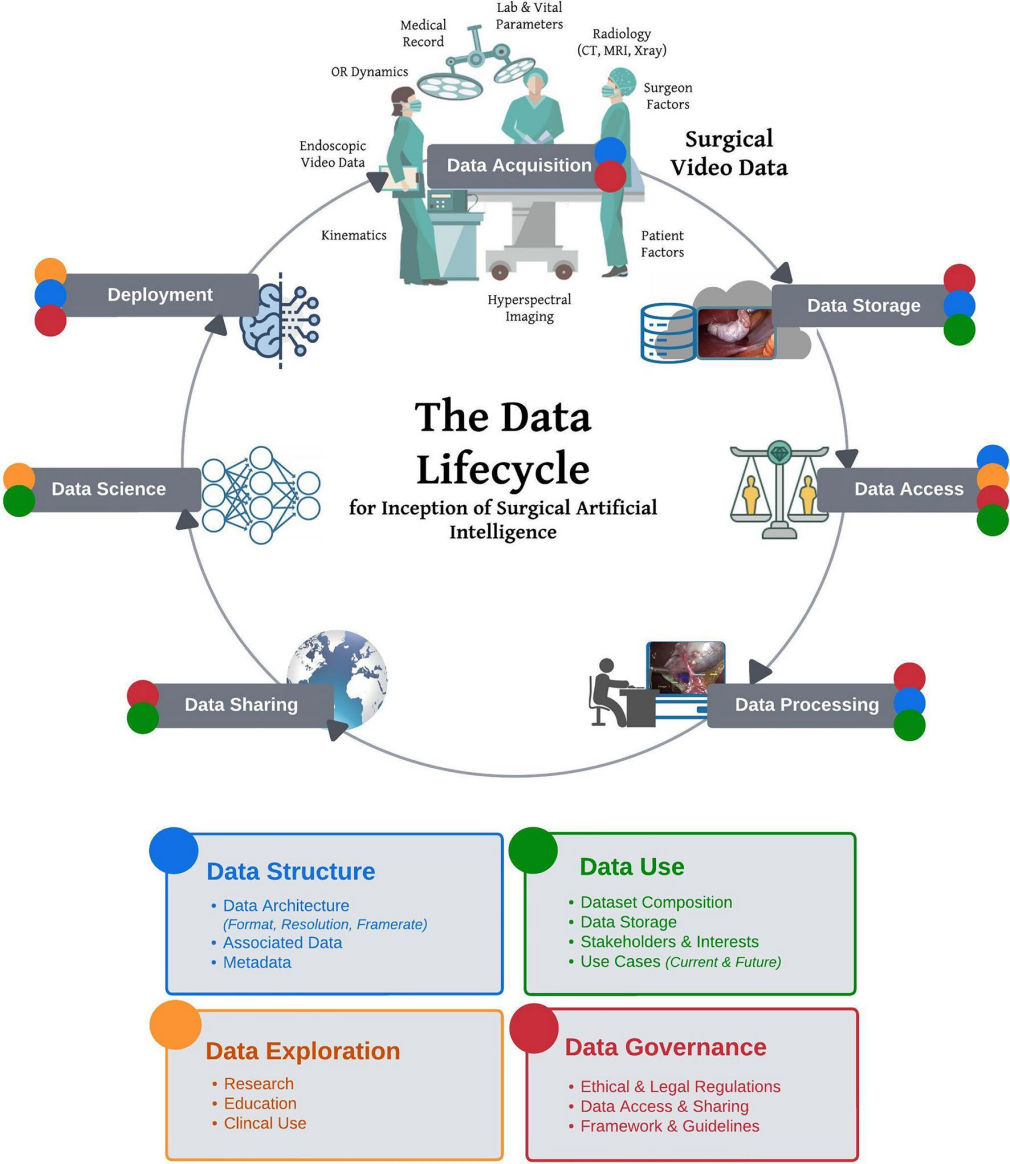
The Data Lifecycle, highlighting stages of surgical video data en route to the creation of AI. Schematic outline of essential attributes of data architecture and infrastructure influencing current data use and future exploration and considerations for adequate governance

In this context, the AI Taskforce of the Society of American Gastrointestinal and Endoscopic Surgeons (SAGES) has developed a pragmatic and strategic plan to foster the development of sustainable and scalable surgical AI, and contribute to the establishment of best practices for efficient and robust surgical data science. The previously published consensus recommendations on surgical imaging and video annotation constitute the foundation for an interdisciplinary framework for adequate labeling and exploration of target features in surgical video data [2]. Additionally, the Taskforce has investigated current commercially available video acquisition platforms for intraoperative video recording [3]. Following these milestones, the SAGES AI Task Force developed the Surgical Video Data Consensus project to address the handling and managing of the multifaceted aspects (Use, Structure, governance, and Exploration) of surgical video data throughout all stages of its lifecycle.

To effectively work with surgical video, a thorough understanding of the multifaceted aspects of data and its management throughout the data lifecycle is crucial for both present and potential future use cases. Meanwhile compliance with legal and ethical principles is paramount. Although data management hurdles have been better addressed in other medical disciplines, such as radiology or pathology, surgical video data management lacks comprehensive guidelines and adequate infrastructure. This shortcoming can be attributed to the myriad of spatial, temporal, and contextual factors that uniquely affect data in the visual domain and their ensuing implications. These factors include patient-related aspects (e.g., anatomy, underlying conditions, organ, and tissue characteristics), surgeon-related elements (e.g., experience, skill, tools utilized), procedure-related factors (e.g., surgical techniques, equipment, intraoperative events, complications), and demographic aspects (e.g., video origin, resource availability, equipment used). Overall, understanding the multifaceted nature of data and its lifecycle has become increasingly important for scientific advancement. Figure 2 depicts the complexity and multifactorial overlap between these 4 key themes quintessentially impacting the value, benefit, and challenges around surgical video data. Key aspects of surgical video data to be considered in the establishment of standardized guidelines are:

- **Data Use** addresses the users and use cases of the data, highlighting the significance of understanding the various interactions between different stakeholders, such as government entities, healthcare organizations, societies, physicians, and patients. Identifying the distinct needs of these users can help inform data management and application strategies.
- **Data Structure** encompasses the architecture and organization of surgical video data and the associated metadata, as well as the processes involved in its acquisition, storage, and distribution. This aspect ensures that data is optimally formatted and readily accessible to various stakeholders for various use cases.
- **Data Exploration** represents the research aspect of data use: investigating current applications, emerging technologies and future potential novel use cases. Data Exploration not only uncovers the potential of the data but also its limitations in the form of biases and unmet needs in numerous fields. This aspect propels innovation and discovery, contributing to scientific knowledge's ongoing growth and evolution.
- **Data Governance** comprises the outlines through which policies, rules, and regulations are formulated and enforced, dictating data utilization and protection across different societal levels. Effective governance fosters responsible data use while addressing privacy and ethical concerns.

**Fig. 2:**
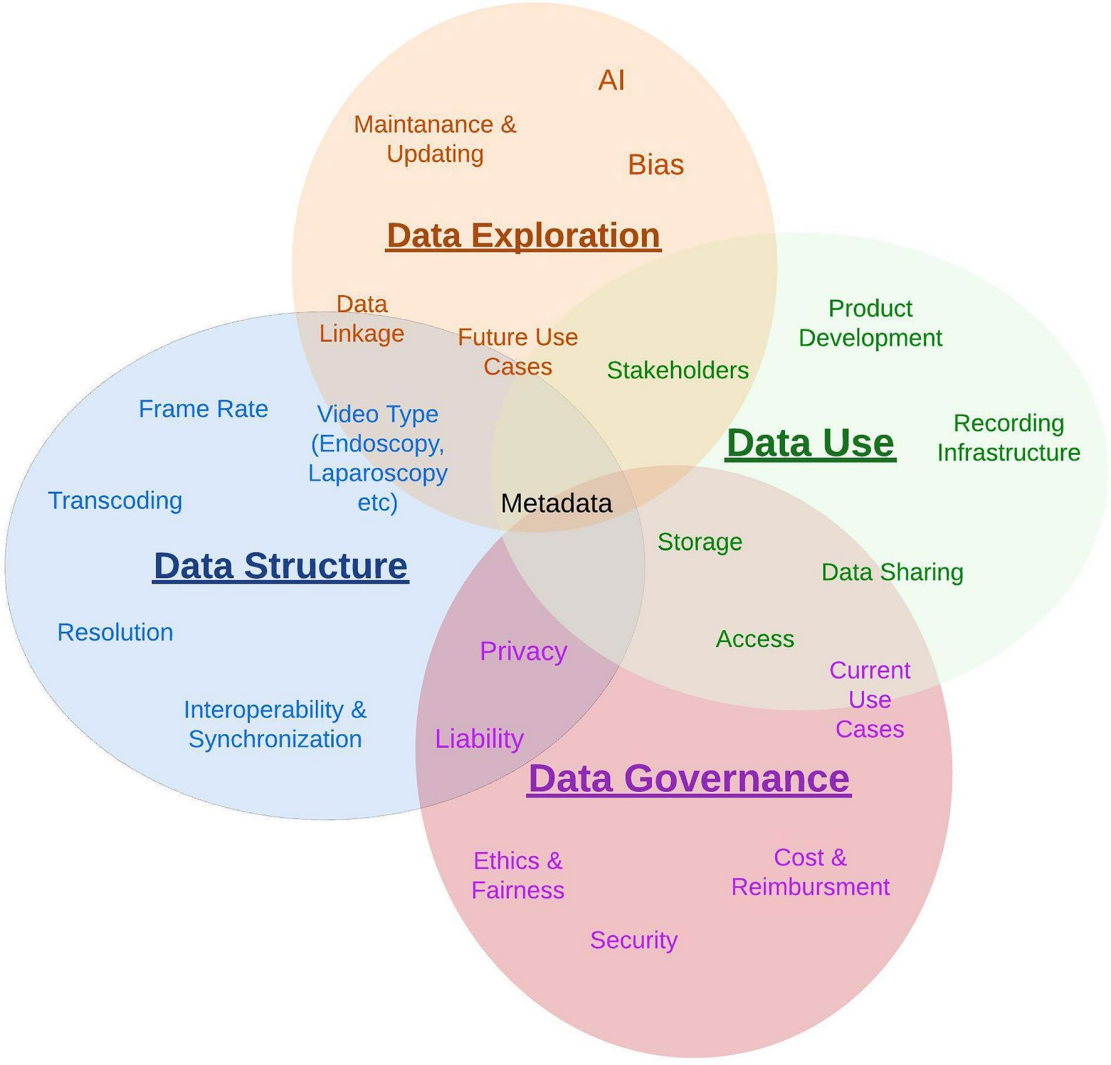
Interdependence of individual aspects influencing all stages of the data lifecycle. At the center of the four key themes impacting surgical video data (data structure, data use, data exploration, and data governance) is the associated metadata. Current and future possibilities arising from data attributes, clinical implications of visual phenomena and outcome related research, traceability and privacy regulations as well as identification and management of bias are fundamentally dependent on the type of available and accessible metadata

## Purpose and scope

The objectives of this consensus project are to (1) determine present-day approaches to managing surgical video data and (2) to suggest guidelines for the essential aspects of data management, with the intention of fostering a more standardized method to improve interdisciplinary and collaborative research efforts. These recommendations are designed to support physicians and engineers involved in the research and clinical application of surgical video-based AI by streamlining data management that enables the comparison of results between research groups, the amalgamation of heterogeneous datasets, and the cross-validation of algorithmic outcomes. The scope of this consensus is confined to surgical video data pertaining to minimally invasive surgery, encompassing laparoscopic, thoracoscopic, endoscopic, and robot-assisted procedures of the chest, abdomen, and pelvis. The objective is to create a foundational framework for surgical video data management to guide the development of more specific methods for organizing, storing, and utilizing surgical video data for training and testing algorithms.

## Materials and methods

### Working group composition, participant selection, and eligibility criteria

A steering group of surgical AI and data science experts was assembled. The subject of video data management for surgical AI was divided into the following four working groups, focused on the domains: (1) Data Use, (2) Data Structure, (3) Data Exploration, and (4) Data Governance. The expert steering group assembled working groups composed of clinicians, engineers and computer scientists from academia and industry. Participants were drawn from the SAGES membership pool and authors of significant works in the domain.

Eligibility criteria for clinician participants were board certification and active practice in general, gastrointestinal, or thoracic surgery surgery or active enrollment in an accredited surgical residency program (Post-graduate Year 3 and above). Additional experience in research related to AI, ML, computer vision, surgical decision-making, minimally invasive surgery (laparoscopic, endoscopic, robotic), or surgical education was required. Engineers and computer scientists had to be actively involved in technical research, focused on surgical data science, AI, computer vision or ML. Completion of a graduate degree was necessary. To acknowledge the industry’s role in the research and development of surgical AI applications, approval from the SAGES Executive Committee of the Board of Governors was obtained to include industry members in the consensus. Industry participation was based on sponsorship of the in-person Data Structure Project and Conference. Each company was permitted to appoint up to two eligible individuals, as outlined below. The appointment of industry participants was reviewed by the chairs of the steering group. Industry participants were required to have a primary role in research and development related to surgical data science and AI and were not allowed to have a primary role in the marketing, sales, or public relations of their company. Non-researchers from the industry, including executives and individuals from marketing and sales without necessary clinical or technical qualifications as noted above, were not eligible to participate in this project. All participants were required to disclose industry ties and potential conflicts of interest. The steering group reserved the right to exclude individuals who reported or exhibited behaviors suggestive of substantial commercial bias and relevant, significant conflicts of interest.

### Working group meetings

Each participant was assigned to one of the four working groups, (1) Data Use, (2) Data Structure, (3) Data Exploration, and (4) Data Governance, taking into account a balanced representation from the multidisciplinary pool of experts. From April 2021 to May 2021, working groups met weekly online to brainstorm and discuss their assigned domains. Each working group was tasked to research relevant literature, discuss findings among the members, and generate recommendation statements within their domain. These statements should address infrastructural needs for surgical video data management and propose standardized methodology for research, education, and clinical applications of the data. In May 2021, each working group presented a summary of their findings via a web conference and suggested recommendations to all members of the consensus working groups. The presentations were recorded to allow members to access the videos for future reference as needed.

### Modified delphi survey

A modified Delphi process was used to evaluate and create a consensus on the recommendation statements resulting from the working group discussions and meetings. The process was performed in two rounds. Participation in the modified Delphi process was contingent on the participation in and reviewing of the final working group presentations. Round 1 was conducted online using Google Forms (Google LLC, Mountain View, CA, USA) in May 2021. Round 2 was held at the SAGES Video Annotation Consensus Meeting in Houston, TX, USA, in June 2021. Attendees could participate in person or via a web conferencing solution. Participants were shown the results from Round 1, and when applicable, discussion, revision, and revoting were held. Voting was performed anonymously using Poll Everywhere (San Francisco, CA).

The a priori criteria for each round of the modified Delphi survey were: (1) ≥ 90% agreement would result in statement adoption with no further revision needed; (2) 80–89% agreement would result in statement adoption but with the option for discussion and revision among in-person attendees; (3) < 80% agreement would require group discussion, revision of the statement, and revote regarding the inclusion of the revised statement during Round 2 of the modified Delphi process.

**Fig. 3:**
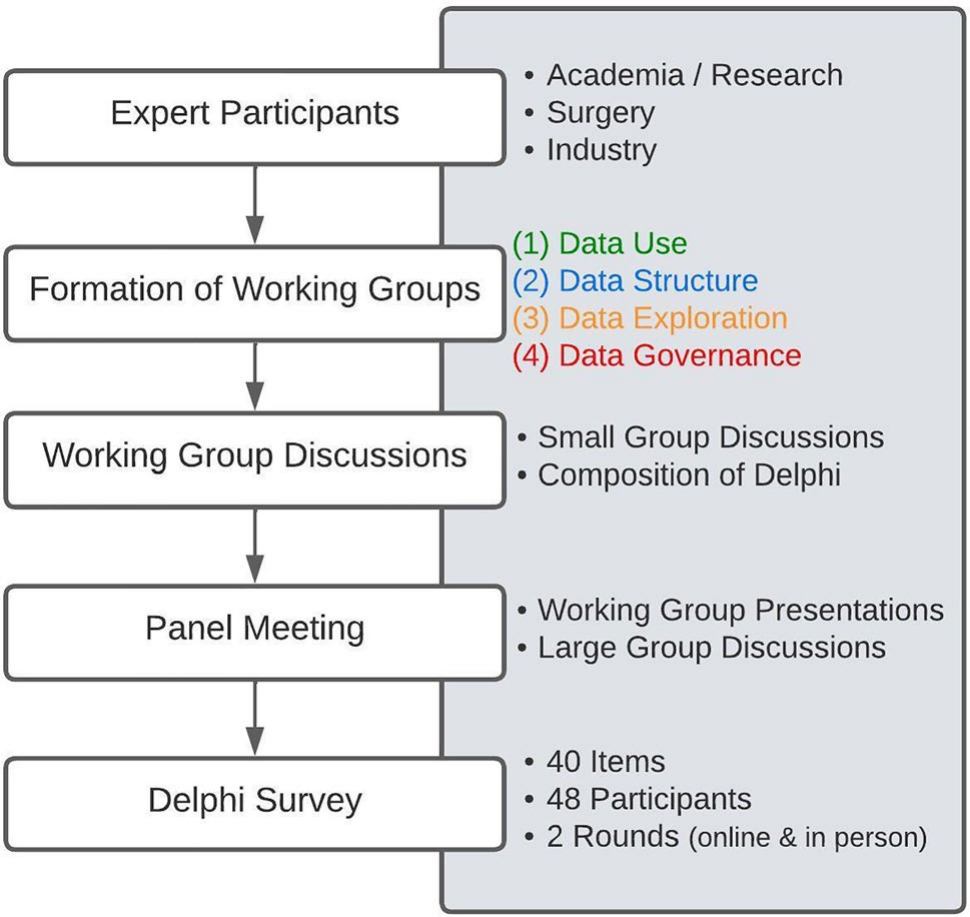
Chronological overview of methodology

Following the completion of the modified Delphi process, the consensus recommendations for each of the four domains were compiled, refining the initial statements based on expert input and votes. The final recommendations, as agreed upon by the expert panel, are presented and discussed below. Figure 3 shows workflow throughout the project.

## Results

### Participant demographics and working group composition

A total of 50 eligible individuals were assigned to four working groups. All participants completed the working group and panel discussions, contributing to the overall consensus process. Both online rounds 1 and 2 of the survey were completed by 48 participants, with no dropouts between rounds. Survey participants consisted of 22 surgeons, 13 engineers or computer scientist, and 13 representatives from industry. Companies represented in the industry panel were Active Surgical, Boston Scientific, Cambridge Medical Robotics, Intuitive Surgical, Medtronic Inc, Olympus, Proximie, Surgical Safe Technologies, Theator and Verily. Academic Representatives specialized in Computer science and Surgery were from the University Hospital London, University of Stanford, University Hospital Cologne, University of Heidelberg, German Cancer Research Center, National Center for Tumor Diseases Dresden, Mount Sinai Hospital New York, IHU Strasbourg, IRCAD Strasbourg, Massachusetts General Hospital, and the Massachusetts Institute for Technology. An overview of the working group members is given in Table 1.

**Table 1:** Overview of working group composition

Chair: Ozanan Meireles, MD FACS Co-chairs: Maria Altieri, MD, MS; Guy Rosman, PhD; Mark Talamini, MD, MBA; and Thomas Ward, MD	
Domain	Working group members
*Data use* pertains to the users and the uses of the data	Leads: Nicolas Padoy, PhD Co-leads: Amin Madani, MD, PhD Members: Pietro Mascagni, Hans Fuchs, Holly Nguyen, Karen Kerr, Justin Collins, Ben Andrew, Brian J Dunkin, Patricia Sylla, Gretchen Purcell Jackson, Bogdan Mitrea, Maria Altieri, Yutong Ban, Quanzheng Li, Amar Chaudhry
*Data structure* refers to the format of the data, including metadata, and the processes of data acquisition, storage, distribution, etc	Leads: Lena Maier-Hein, PhD Co-leads: Pierre Jannin, PhD, Keno März, PhD; Carla Pugh, MD, PhD Members: Anthony Jarc, Filippo Filicori, Imanol Luengo, Beat Müller, Tina Chen, Danyal Fee, Thomas Ward
*Data exploration* addresses the research aspect data use. Current uses, potential novel applications, unmet needs, etc	Leads: Danail Stoyanov, PhD Co-Leads: Mehran Anvari, MD; Guy Rosman, PhD Members: Dotan Asselmann, Lee Swanström, Steven Bishop, Saeed Latif, Marissa Crosetti, Serena Yeung, Daniel Hashimoto
*Data governance* refers to the policies, rules, regulations, and oversight at different levels that influence the use of the data	Leads: Stefanie Speidel, PhD Co-Leads: Paresh Shaw, MD; Elan Witkowski, MD Members: Silvana Perretta, Steven Schwaitzberg, Chris Boyle, Mark Thalamini, Martin Wagner, Peter Kim, Hirenkumar Chandraant Nakawala, Robin Blackstone, Sanjeev Dutta, Orleigh Bogle, Swaroop Vedula

### Consensus statements, recommendations, and discussion

Through the working group discussions and modified Delphi process, consensus recommendations were achieved for each of the four themes: Data Use, Data Structure, Data Exploration, and Data Governance. These recommendations provide guidance on the best practices for managing surgical video data, ensuring its quality and applicability for various stakeholders in research, education, and clinical practice. Discussions centered around core themes pertaining to surgical video data, such as associated metadata, data storage, stakeholders and bias, were found to exhibit redundancy across working groups. Particularly the topic of bias was intensely discussed in the ‘Data Structure’ and ‘Data Exploration’ working groups. Consequently the results were condensed to eliminate repetition.

### Data use

#### Data acquisition and dataset composition

**Fig. 4:**
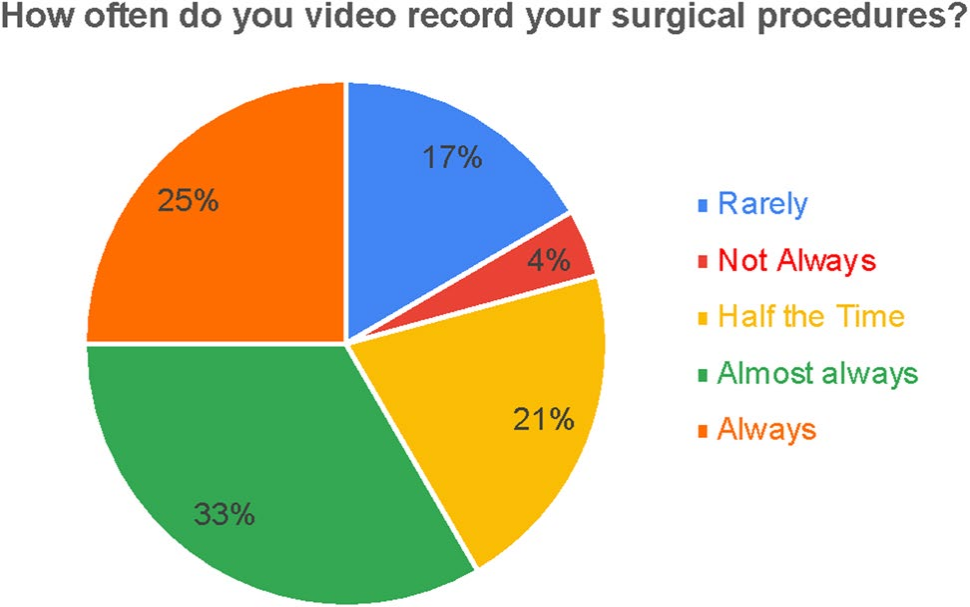
Results of statement 5—frequency of recording among surgical survey participants

- Statement 1: “As of May 2021, it is difficult to perform multi-institutional studies involving surgical video due to the lack of well-defined data structure standards.” (95,9% Strongly Agree or Agree.)
- Statement 2: “Should all surgical procedures be recorded?” (81.3% Strongly Agree or Agree)
- Statement 3: “Should relevant surgical procedures, such as rare cases, events, new techniques, be stored?” (81.8% Strongly Agree or Agree, 20.8% Neutral)
- Statement 4: “Data should be collected holistically for future scientific use” (91.7% Strongly Agree or Agree)
- Statement 5: “How often do you video record your surgical procedures?” (See Fig. 4)

#### Key points

(A) Challenges and implications of data acquisition
• The lack of well-defined data structure standards makes it difficult to perform multi-institutional studies involving surgical video.
• Holistic data acquisition raises infrastructural, logistic, and legal questions.
• Concerns around the sustainability of holistic data acquisitions include structural characteristics of the data and the type and duration of data storage.
(B) Importance of data diversity
• Flexible, less dogmatic data acquisition policies could facilitate video recording in the operating room.
• Recording less frequent, more complicated cases ensures representation of rare events and intraoperative complications.
• The abundance of research applications focused around routinely performed procedures is high; the additional value of recording such cases in large quantities is questionable.

Aside from the SAGES AI Taskforce, various other collaborative, interdisciplinary initiatives have been formed, with the goal to standardize surgical data science. Standardized frameworks and guidelines for using surgical video data aim to ensure inclusivity, diversity, useability and ethical and legal soundness of surgical data sets [3, 4]. Holistic data acquisition refers video the recording of surgical procedures from start to finish, recording of all or the majority of performed procedures, as well as the collection of other associated imaging and video data (e.g., intraoperative endoscopy, preoperative imaging data), and documentation of demographics and metadata associated with the case.

While holistic recording of all performed procedures would be ideal to ensure large dataset reflecting adequate diversity, it raises infrastructural, logistic and legal questions. Concerns around sustainability of holistic data acquisitions include a) structural characteristics of the data (file size, quality, format, type of procedure, contained PHI) and b) type and duration of data storage. In practical terms storage capacity is a highly relevant limiting factor in surgical video data acquisition, as servers are expensive resources, and maintenance of and accessibility to the data add to the cost. Mandating recording, storage, and maintenance of all performed procedures as the standard, in an ‘all or nothing’ principle, may discourage data acquisition at resource-challenged institutions. This could limit datasets to recordings from select, well-resourced medical centers with sufficient available means for acquisition, storage and computation and discourage centers with less established recording infrastructures. Rather than encourage data diversity, this would lead to a significant collection bias and limit diversity of origin of the data.

Arguably, flexible, less dogmatic data acquisition policies could facilitate video recording in the operating room and hence support recording efforts. Less dogmantic recommendations for data acquisition will increase overall data abundance, which will increase abundance of less frequent, more complicated cases and ensure representation of rare events and intraoperative complications. Thus datasets would allow for more comprehensive analysis of the overall surgical workflow and provide useful information for future applications and use cases. Currently existing, publicly available datasets predominantly cover routinely performed procedures, while less frequently performed procedures are not represented well in large scale dataset [5]. Therefore the abundance of research applications focused around these routine procedures is high, and, the additional value of recording such cases in large quantities is questionable. Also, while recording of every case has the potential to obtain more inclusive datasets and better coverage of rare scenarios, too much identical data may add irrelevant ‘noise’ to datasets.

Overall, the availability of collected data suitable for education, research, and clinical quality assurance is still low, therefore aiming for holistic data acquisition of surgical video data – meaning collecting as much data as possible—should be a priority. However, considerations around collecting everything and resulting tradeoffs in data quality, relevance to the dataset and practicality have to be made on a per-institution level. The resulting overabundance of data will require filtering out and deletion of redundant recordings to obtain an overall balanced dataset between frequent and rare samples. But surely incentivizing and facilitating recording of surgical procedures in general will obviously add value to the field. Moreover, exchange of surgical video data presents an essential prerequisite for large and diverse datasets, specifically with respect to origin of the data. Arguably, adequate coverage of low-probability cases and events, as well as inclusivity of data from resource-challenged and remote origins is only achievable through data exchange and multi-institutional collaborative dataset composition. Local divergence in governing regulations and different technical premises for data recording, processing, and storage present the main obstacles for data sharing. The lack of transparency in regulation and governance of storage and management of surgical video data and the associated Personal Health Information (PHI) currently impedes multi-institutional studies. Additionally current and future capabilities for exploring this data have to be regulated appropriately before large scale data exchange can occur. Unrestricted use and distribution of the data for potential future use cases may be regarded as ideal for research and innovation but conflicts with principles of data ownership and governance.

#### Stakeholders and use cases

**Fig. 5:**
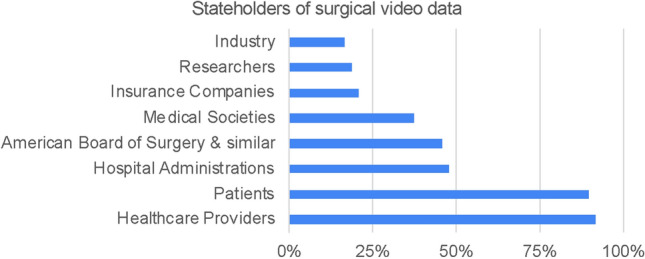
Results of statement 6—relevant stakeholders in surgical video data, as identified by survey participants

**Fig. 6:**
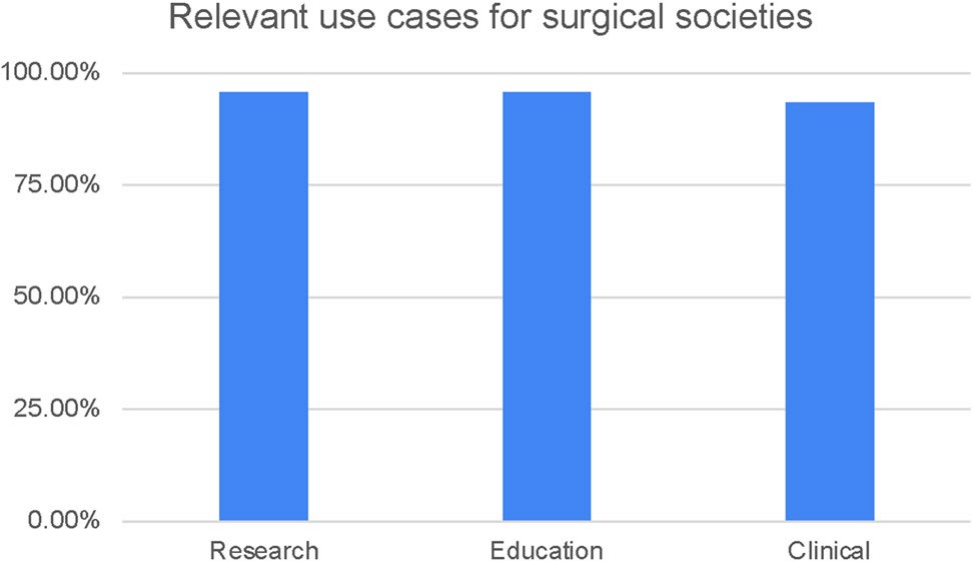
Results of statement 7—almost all survey participants identified research, education, and clinical use cases of surgical video data as relevant to surgical societies, such as SAGES

**Fig. 7:**
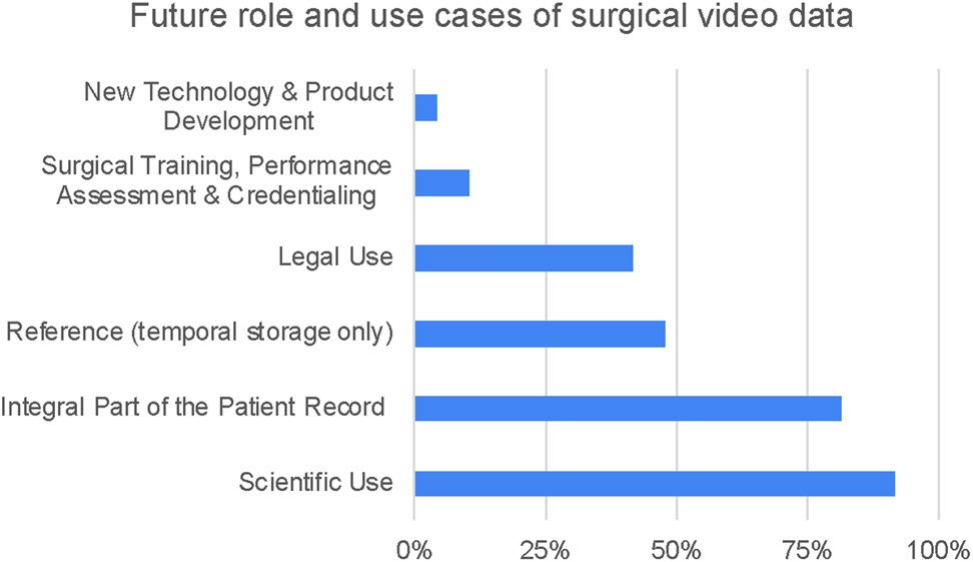
Results of statement 8—future applications and use cases of surgical video data, identified by survey participants

- Statement 6: “Which stakeholder has the right to access recorded surgical videos?” (See Fig. 5)
- Statement 7: “For a surgical society, such as SAGES, how relevant is the use of video data for A) research B) education C) clinical purposes?” (See Fig. 6)
- Statement 8: “Which role will surgical videos take on in the future?” (See Fig. 7)

#### Key points

(A) Stakeholder roles and access
• Healthcare providers and patients should have primary access to surgical video data.
• Researchers and industry were ranked lower in terms of access to surgical video data.
• Individual, specific purposes of access should be carefully evaluated and restricted to prevent misuse.
(B) Use cases and future implications
• Current applications range from computer vision and AI to performance evaluation, skill assessment, and virtual reality.
• In the future, surgical video data could be used for accreditation, licensing, medical reimbursement, and legal investigations.
• Interaction between different stakeholders is key for adequate use of the data and to ensure clinical benefit.

Stakeholders are defined as people or entities with interest or concern in something. With respect to surgical video data, that interest depends on the context of use of the data and the associated metadata. Most survey participants agreed that healthcare providers and patients should have primary access to surgical video data (91.7% and 89.6%), followed by hospital administrators, board institutions, such as the American Board of Surgeons and medical societies (47.9%, 45.8%, 37.5%). In comparison, researchers and industry were ranked lower (at 37.5%, 18.8%, and 16.7%, respectively). While there is notable overlap between the stakeholders in surgical video data identified in this study and previous investigations [4], expert panel discussions following this survey shifted from stakeholder roles to a focus on use cases. While surgical video data is undeniably being used for research, education, and clinical practice already, these terms are very broad and should be further specified. More specifically, current applications range from computer vision and AI, over performance evaluation, skill assessment and virtual reality, to retrospective analysis of complications and surgical techniques [6] and many more. In the future, surgical video data could even be used for accreditation and licensing, medical reimbursement and legal investigations, such as malpractice lawsuits [7, 8]. Therefore two main considerations were highlighted around data use by different stakeholders: infrastructure of data storage and access and governing regulations.

As all of the listed entities and stakeholders have various interests in using the data, the individual, specific purpose of access should be carefully evaluated and restricted to prevent misuse. From a healthcare perspective, any data related to the patient, to procedures and to clinical outcomes, including video and images, should first and foremost be used to promote personalized care, best practices and ultimately benefit the patient. Thus legal frameworks, such as HIPAA and GDPR, mostly prohibit access of the data without a purpose related to patient treatment. Currently, this means institutional stakeholders only have unrestricted access to the video data through specific consent. As it stands today, however, researchers and administrators of the data have less restricted access, while patients may have no access at all. This discrepancy in access and use of the data has to be addressed accommodating all stakeholders’ perspectives and should be focused on more in depth in future studies. All stakeholders should be equally aware of the implications of data existence, data management and data use. At the moment, this awareness is most evidently lacking in patients among other entities. Providing patients with more information about their data and their patient record, authority and rights over the data, as well as education about how to exercise that their rights, will empower them to make informed decisions. If surgical video data is included as part of the patient record, it can also be used to educate the patient about their disease and current and future treatment. On the other hand, storage of surgical video data as part of the patient record can implicate the use of surgical video data for legal purposes, which may be regarded as a ‘double edged sword’ and hence reduce collection. Regulations protecting surgeons and physicians have to be put in place to incentivize video recordings in the operating room. While surgical video data is currently not routinely consulted as evidence in malpractice lawsuits, it more likely than not could exonerate the surgeon rather than burden.

Infrastructurally, long-term storage of surgical video data as an integral part of the patient record and use for scientific purposes were identified as most relevant (81.3% and 91.7%), followed by short-term storage only and legal use (47.8% and 41.7%). As research and innovation progresses, additional use cases for surgical video data emerge. To account for future possibilities arising from the data, the short-, intermediate-, and long-term applications, both clinically and technically, need to be outlined. While we may not know how to implement certain ideas and concepts based on surgical video data yet, ‘future proofing’ the data, both in terms of structure, use and storage is important. Acquiring the data in a holistic fashion therefore presents an essential prerequisite for new use cases. Novel technological methodologies, that are currently being developed, already give an indication of future uses of the data. But these insights into future purposes of the data are predominantly gained in research and industry rather than clinical practice. Additionally, translating scientific progress into clinical practice will require surgical video data to be accessed by clinicians, and researchers and industry alike. Interaction between different stakeholders is key for adequate use of the data and to ensure clinical benefit resulting from it. Medical societies may unite multi-stakeholder interests, by promoting use of surgical video data for research, education, clinical use cases as well as innovation and development of new technologies. All three use cases rely on video of surgical procedures to document and foster good and bad clinical practice examples. Insights gained from video data can be more easily and widely disseminated within large scale organizations such as medical societies. Furthermore, medical societies can provide an infrastructure for data management (e.g., guidance on data format, storage, access, regulatory considerations) and a democratic platform for data sharing, while promoting the mission to use the data for improvement of clinical care, research, and education equally. While these societies do not hold jurisdiction, they do have a regulating effect and can provide transparency of regulatory and structural aspects to be considered throughout the data lifecycle. To enable future use of the data, structured storage of the data is required. Currently there is no standard for storage duration and modality. Use case specific indexing of the data, editing and categorizing the data into procedures, keys steps, intraoperative events, milestones, and decisions could offer significant value to surgical training and performance assessment.

### Data structure

#### Format and architecture

- Statement 9: “Videos should ideally be stored in their source format, with future transcoding minimized to avoid loss of visual and audio data.” (81.5% Strongly Agree or Agree)
- Statement 10: “When feasible, a standardized and configurable data architecture is favorable to a specific and curated architecture in order to improve interoperability.” (> 81% Strongly Agree or Agree)

#### Key points

(A) Standardized data architecture and challenges
• Standardized data architecture facilitates interoperability across the data lifecycle.
• It can improve taxonomy and interdisciplinary discourse.
• However, standards must accommodate various use cases and factors, balancing the needs of AI, surgical education, and clinical evaluation.
• Strict standards may limit data diversity and increase collection bias.
(B) Quality requirements and trade-offs
• Quality requirements depend on the use case, with higher resolution needed for model development than teaching.
• Higher quality data requires more storage, potentially limiting access for resource-challenged sites.
• Storing videos in their original format may conflict with governing regulations and raise practicality and sustainability concerns.

Predefined standards for data architecture would facilitate interoperability for all stages of the data lifecycle, from data acquisition to storage, sharing, and use. Furthermore standardized data architecture could lead to a more standardized taxonomy and improve interdisciplinary discourse. On the other hand, such standards would have to be applicable to all use cases of the data and account for a variety of factors. Data architecture suitable and required for ML and AI purposes are not necessarily favorable for innovation and development of new technologies, or surgical education, training, and clinical evaluation. Despite the clear advantage of high resolution data, the quality requirements of the data depend on the use case. Videos intended for model development require have higher frame rates and resolution than videos used for teaching purposes. Furthermore higher quality data demands more storage, again restricting sites with less access to appropriate server capacity. While higher resolution videos and images may always be regarded as containing more information, hence more valuable, low resolution data should not be disregarded. Strict, predefined standards may contradict with inclusivity of data that does not fit these standards. While recording surgical procedures should aim for high quality standards, which are yet to be established by guidelines, a strict adoption of criteria linked to format, resolution and frame rate could prevent data acquisition in resource-challenged sites and ultimately limit data diversity and increase collection bias. Moreover, storing videos in their original format conflicts with governing regulations and raises questions about practicality and sustainability. While the original format offers multi-purpose use of the data, removal and separate storage of PHI, audio data and out of body images is important to comply with privacy law regulations.

Ultimately, reasonable recommendations for video data architecture, with respect to format, resolution and frame rate should be proposed. The above statements clearly emphasize that a standardized, configurable data architecture, which allows for various ways of transcoding, is recommended. The specifications of that data architecture have to be outlined further and should aim to account for different use cases of the data, practicality, compliance with legal regulations and overall incentivize the recording of surgical procedures in general.

#### Integration of other data modalities

**Fig. 8:**
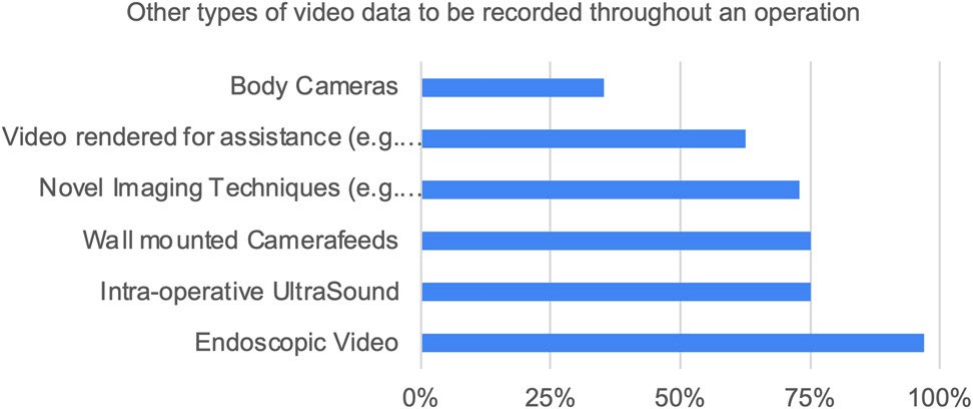
Results of statement 12—other types of video data to be recorded, besides intraabdominal video footage

- Statement 11: “Synchronizing different data sources inside the OR with surgical video streams should play a crucial role for research in the future.” (93.7% Strongly Agree or Agree)
- Statement 12: “Which other types of video and imaging data (if any) should be recorded over the course of the whole operation?” (See Fig. 8)

#### Key points

- Combining various data sources in the OR can enhance the understanding of surgical workflow and facilitate best practices and technology development.
- Interoperability and access are currently limited by distinct systems, restricted access, and technical requirements.
- To enable multimodal data integration, open accessibility standards must be established, and synchronization details must be explored.

With increasing digitalization of medicine, more data provides insights on different angles of patient care. Possible data sources in the operating room include medical records, vital parameters, kinematic and telemetric data and audio recordings. Aside from intraabdominal, surgical video footage of the procedure itself, endoscopic video, intraoperative Ultrasound and even transcripts of OR dynamics (e.g., video recordings of the operating room itself) provide valuable information to the surgeon, which they consult for intraoperative decision making. Never imaging modalities add information about blood flow in real-time (hyperspectral imaging) [9] and can project visualizations of anatomy and pathology onto the operating field (augmented reality) [10]. The type of data recorded depends on the use case of the data. While there are various clear use cases for endoscopic data at the moment [5, 11–13], other modalities of video and imaging data may also be useful for improving clinical outcomes in the future. For example, the suspected link between team dynamic in the operating room and patient outcomes can only be properly analyzed with video and audio footage covering the operating room itself.

Acquisition of multimodal data and synchronization with intraabdominal video data is currently limited, due to limited interoperability between systems. Additionally, restricted access to the data and low abundance of technical requirements for acquiring, storing, and synchronizing such data limit interoperability. At the moment, the various data modalities are mostly stored separately from each other, if acquired in the first place. To enable multimodal data integration, adequate data infrastructures have to be established and device manufacturers would have to share access and establish open standards of accessibility for distinct purposes, such as research. Secondly, technical details of synchronization have to be explored, which includes accuracy and precision of synchronization. Synchronizing robotic telemetry to procedural video may require millisecond precision, whereas linking patient vitals to administration of intra-operative medicines may only require second accuracy.

Overall, to comprehend surgical workflow retrospectively and remotely, synchronization of all sensory information available to the surgeon in real-time adds value and detail to the surgical video data. Understanding surgical procedures from a variety of different angles could be paramount to the implementation of future best practices and assist the development of new technology for the operating room. Yet recording, processing, and manipulating such data opens the discussion for other governance issues.

#### Metadata

**Fig. 9:**
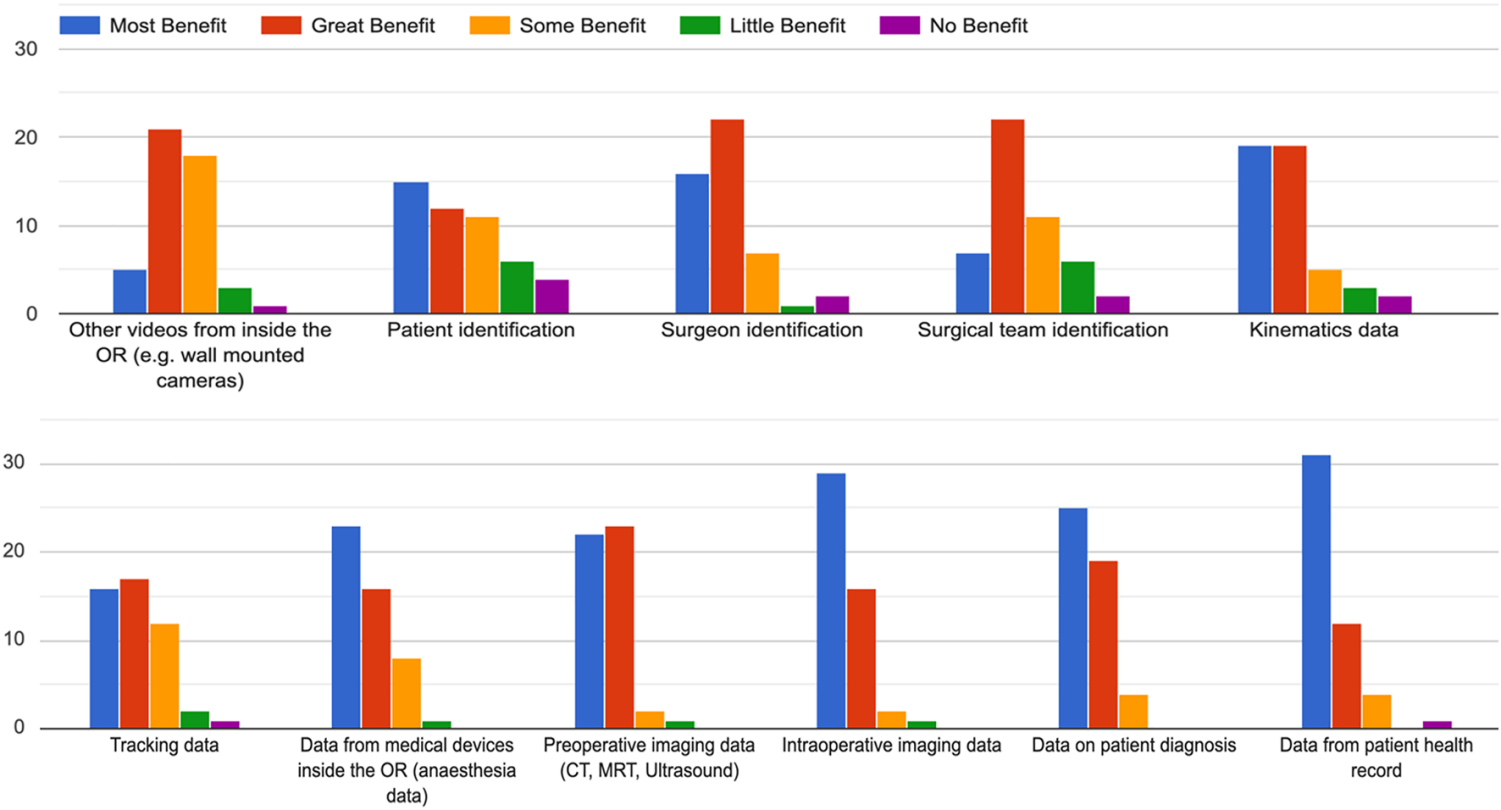
Results of statement 15—metadata that would provide most benefit if linked to surgical video data, as identified by survey participants

- Statement 13: “Videos should be able to be linked to rich metadata, such as acquisition location, surgeon, and patient data.” (89.6% Strongly Agree or Agree)
- Statement 14: “In terms of prospective data collection, which one would you I find more beneficial?” (83.3% Holistic collection data for future scientific use, 16.7% minimal data for planned analysis)
- Statement 15: “The following metadata would provide the most benefit if linked to surgical video recordings:” (See Fig. 9)
- Statement 16: “Metadata regarding access rights and extend should be collected to enable additional use cases (i.e. Who has accessed the data at what time? Who is eligible to access the data under which circumstances?)” (93.8% Strongly Agree or Agree)
- Statement 17: “Metadata regarding hospital governance and privacy should be collected to enable additional use cases (i.e. How and under what circumstances is data currently stored / shared / managed?)” (93.7% Strongly Agree or Agree)

#### Key points

(A) Metadata and governance concerns
• Rich metadata expands potential use cases and provides essential context.
• Access privileges should be determined based on stakeholder type and purpose.
• Information about metadata access and use can help prioritize data storage and maintenance.
• Defining access rights and modalities can improve oversight, interoperability, and privacy protection.
(B) Current challenges and future recommendations
• Standardized control mechanisms for surgical video data and metadata access are needed.
• Linking intraoperative video to patient outcomes can maximize data potential for improving surgical care.
• Patient consent and willingness to provide metadata may vary.
• Future regulations should involve all stakeholders in data creation, distribution, and handling, as well as law and policy makers.

Metadata probably constitutes the biggest concern around the use and governance of surgical video data. While clearly reflected in the results of this survey, more metadata is beneficial, as it expands the potential current and future use cases of surgical video data and provides essential context for clinical outcome research and progress in best practices. Yet, not all metadata may be useful in every context and differential access privileges should be established based on stakeholder type and access purpose. Information regarding access and use of the metadata can provide insight into what metadata is relevant for current and future use, therefore narrowing down what data should be stored and maintained. Defining access rights and modalities to metadata can assist the various stakeholders to better oversee the available data, determine what can and should be done with the data, and ultimately enable interoperability. Furthermore, access information can support appropriate governance of data access by uncovering the gaps and areas of improvement for privacy protection.

Currently no standardized control mechanisms are in place to determine who can access and use surgical video data or the associated metadata, nor for what purpose and which duration access is granted. HIPAA and GDPR provide definitions of which data falls under PHI, yet the clear categorization of surgical video data, especially when metadata and out of body images are removed, remains to be clearly defined. And while most stakeholders in surgical video data perceive rich metadata to be beneficial, they also agree that existing regulations largely impede research and progress [4]. For long-term and efficient use of surgical video data certain metadata is essential, particularly when investigating the link between patient outcomes and visual phenomena in surgical video data. Linking intraoperative video to patient outcomes, complications and near misses maximizes the data potential to improve surgical care. While in most institutions patients consent to having their minimally invasive procedure recorded as part of their patient record, to be used for research, education, and clinical purposes, patient consent and willingness to provide associated metadata may vary. For the establishment of future regulations and recommendations of surgical video data and the surrounding metadata it is, therefore, important to a) investigate how this data is currently handled, b) outline drawbacks in these current practices, c) involve everyone involved in the creation, distribution and handling of the data as well as d) law and policy makers. In conclusion, this expert panel advices recording of rich metadata alongside surgical video data, alongside statistics and contextual information around access and use of the metadata to account for adequate protection of privacy.

### Data exploration

#### Bias

The topic of bias within surgical video data and AI systems was discussed extensively in the working groups focused on ‘Data Structure’ as well as in the working group focused on ‘Data Exploration’. The following findings reflect the results from both independent working group discussions and their joined discourse.

**Fig. 10:**
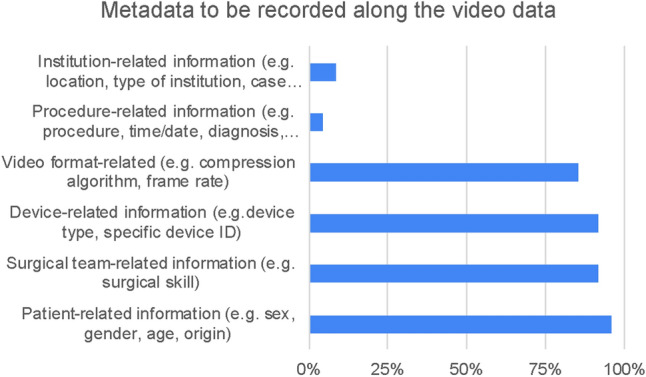
Results of statement 20—type of metadata, that should be recorded alongside surgical video data to avoid bias

- Statement 18: “Unawareness of biases potentially leads to systematic over- and underestimation of algorithm performance.” (91.7% Strongly Agree or Agree)
- Statement 19: “Studies in the broader field of biomedical image/video analysis should take potential biases within the data adequately into account when training and validation AI algorithms” (87.5% Strongly Agree or Agree)
- Statement 20: “To avoid biases, the following metadata should be stored along with video data:” (See Fig. 10)

#### Key points

- Addressing bias in surgical video data is crucial for developing fair and inclusive AI algorithms.
- Experts stress the importance of metadata and contextual information in identifying and reducing biases, provided that appropriate regulations and infrastructures are in place.

Selection and confounding bias were previously identified as particularly influential to surgical datasets and the technology resulting from the data [14, 15] and one may inflict the other. As it is difficult to understand and comprehend bias without contextual information about surgical video data and associated metadata, it is unsurprising, that existing publicly available surgical video datasets mostly do not account for biases at all. However, most current research in surgical AI is either based entirely on publicly available datasets, or references to methodologies based on publicly available data as best practices. While not all metadata may be required to account and reduce bias, particularly information related patient and surgeon factors were identified as crucial to reduce bias. Additionally geographical origin of the data can provide insight to surgical practices (e.g., surgical approach/technique used; devices available; hospital culture). As of now, unrestricted access to this information contradicts existing privacy laws, which in turn results in an unknown bias in public datasets. Recommendations related to dataset compositions, particularly public dataset composition, should promote data diversity and allow for identification and reduction of bias within the dataset. Information about demographics of publicly available datasets would significantly advance the field and assure more fairness and inclusivity in the resulting technology. It was noted that the tradeoffs between upholding privacy concerns and catering to bias in future tasks has a tradeoff and will require careful consideration [16].

As previously discussed, the data structure and use section of this paper, high quality standards with respect to data structure are favorable to ensure a wide range of usability of the data. Yet, these standards may exclude data from resource-scarce medical centers, which in turn limits data diversity and results in further bias. More importantly standardized guidelines for data exchange and transparent governing regulations should be put in place to support large, diverse, and inclusive datasets. This survey highlights that bias negatively impacts algorithmic performance, and current practices in surgical AI do not appropriately address bias. Bias can be identified and addressed through metadata and contextual information around the video data, but appropriate regulations and wide spread infrastructural resources need to be established to enable holistic data and metadata collection.

#### Future research, emerging technologies, and AI

- Statement 21: “To support future research, education and clinical quality improvement, we need to support numerous data sources, including aggregation of data from multiple institutes and synchronization of multimodal datasets.” (97.8% Strongly Agree or Agree)
- Statement 22: “Datasets need to be open research studies not just explicit pre-conceived development of specific capabilities.” (> 80% Strongly Agree or Agree)
- Statement 23: “To support future research, data exploration should enable both improvement of existing technical capabilities and AI models, as well as, extension towards and development of new capabilities.” (97.9% Strongly Agree or Agree)
- Statement 24: “To support future AI applications, we need to reduce entry barriers in terms of data privacy, coverage of surgical data within end-to-end patient care, and ease of matching novel technical capabilities to actual clinical needs.” (93.3% Strongly Agree or Agree)
- Statement 25: “Exploration of potential use of AI in clinical practice, should involve exploration of human factors to understand product utilization and value.” (95.5% Strongly Agree or Agree)
- Statement 26: “Exploration of AI in surgical video can lead to improved outcomes and new insight into rare events/patterns/disease that is not visible to the general surgeon due to infrequent occurrence (human overfitting).” (97.8% Strongly Agree or Agree)

#### Key points

- Future Research: The expert panel agrees on the need to support numerous data sources, including aggregation of data from multiple institutes and synchronization of multimodal datasets.
- Emerging Technologies and AI: The panel emphasizes the importance of reducing entry barriers regarding data privacy, covering surgical data within end-to-end patient care, and matching novel technical capabilities to actual clinical needs.
- Clinical Relevance: Data acquisition should be driven by clinical necessities while also considering the exploratory nature of research.

Exploration of surgical video data, the associated metadata and other data modalities linked to the surgical video data, raises two main questions: what is technically achievable with the data and what capabilities are clinically desirable? Currently hypothesis-driven use of the data mostly involves prospective data collection and is limited to the type of clinical task and labels in focus, while data driven approaches often lack clinical relevance. In other words should a clinical problem or research question inspire data collection or should the available data inspire research questions? Similar to the chicken and the egg analogy, both approaches most likely have some degree of validity. The overall goal of surgical data science is the improvement of surgical therapy with a clinical benefit to the patient, meaning clinical necessities should predominantly drive data handling. Yet narrowing data acquisition parameters down to requirements for preconceived use cases would mean closing our minds to future capabilities and contradict the exploratory, curious nature of research.

The community should be mindful of emerging trends in AI, that affect the way we view the importance of data and annotations. Examples include self-supervision [17] and reinforcement learning techniques, as well as interactive, closed-loop systems, which display their own data challenges. From another perspective, the inherent risk of surgery, and advances in alternative treatments may affect the set of clinical tasks where surgical AI is most required. Innovations in natural language models and the role of foundation models in both computer vision and natural language processing may change how we value, and therefore use, data in the future. Yet, even with increasingly sophisticated foundational models on the uprise [18, 19], that are trained with large amounts of data but a reduced set of detailed labels for novel tasks [20, 21], the challenge of validation for complex models remains, and requires high-quality, well-curated data. While foundation models can achieve novel capabilities that resemble general artificial intelligence, they may fail in subtle and unpredictable ways^22^, making it essential to validate and test these models on diverse and representative datasets, to ensure that they generate generalizable, accurate, and fair results.

### Data governance

#### Privacy, traceability, and consent

- Statement: “To support future ethical AI applications, data management should be traceable allowing patients to revoke rights to their records” (> 80% Strongly Agree or Agree)
- Statement: “To support future AI applications, data extraction should support better traceability of the source, selection, and processing of the data, so that we can prevent biases and artifacts between ML training and deployment.” (88.9% Strongly Agree or Agree)

#### Key points

- Ensuring data traceability while maintaining privacy and anonymity is technically and legally challenging.
- Allowing patients to revoke consent at any time could overcomplicate data acquisition efforts and impede technological progress.
- Educating patients about the use and implications of their data could empower them to make better-informed decisions and potentially reduce the need for secondary removal of consent.

It is both technically and legally challenging to ensure traceability of data back to its origin, while complying with privacy laws and maintaining patient anonymity. Practically this would mean adequately deidentifying and anonymizing the data, while separately storing a key to reidentify the data. Standardized guidelines for data structure and detailed instructions on how to store video data adequately, promoted by medical societies, could facilitate that. But mandating data storage that allows for secondary traceability for the purpose of allowing individuals to revoke consent may in turn overcomplicate things and decrease data acquisition efforts. While a certain level traceability would allow for retrospective removal of consent, the question arises until when in the data lifecycle patients should be allowed to remove consent which in turns highlights issues around data ownership and access rights. Advocating for patients authority over their data and educating them further about use and implications about their data would empower patients to make better-informed decisions and may reduce the need for secondary removal of consent overall. For example, should individual patients’ data be removed from finalized technology after significant time, resources and money was allocated to the development of this technology and potential benefit for greater community results from it? For commercially available technology, which is based on large scale datasets, it may be near impossible to do so and would significantly impede technological progress. Undoubtably more insight into data origin and other changes made to the data along its lifecycle would help to identify and address bias within large datasets. We would like to refer to the previous section of the manuscript dedicated to bias.

#### Stakeholder-specific data use

- Statement 24: “Patients may use surgical video data for multiple reasons, including for education, to inform consent, and for documentation of their care” (> 80% Strongly Agree or Agree)
- Statement 25: “Surgeons and researchers, may use surgical video data for education, documentation, assessment, research, scientific communications, legal evidence and self-promotion.” (86.6% Strongly Agree or Agree)
- Statement 26: “Scientific societies, may use surgical video data for research, education, credentialing, accreditation, advertising and visibility” (82.3% Strongly Agree or Agree)
- Statement 27: “Healthcare institutions use or may use surgical video data for workflows optimization, resource allocation, auditing, quality improvement, legal evidence and advertising.” (87.6% Strongly Agree or Agree)
- Statement 28: “MedTech companies use or may use surgical video data for research, development, assessment, regulatory approval, post-market monitoring and marketing.” (> 80% Strongly Agree or Agree)
- Statement 29: “Health insurances use or may use surgical video data, as aggregated data, not individual data regarding patients and surgeons, for risk assessment, pricing, reimbursement and legal evidence (> 80% Strongly Agree or Agree)
- Statement: “Public institutions such as governments, regulatory and credentialing bodies use or may use surgical videos for research, education, develop and assessment of policies, and credentialing.” (88.4% Strongly Agree or Agree)
- Statement: Surgical video data primarily collected by a stakeholder for a given use may be accessed by multiple stakeholders for other uses, giving that proper agreements and permissions are in place (> 80% Strongly Agree or Agree)

#### Key points

- Stakeholder-specific Data Use: Data access should be use-case specific, consistently favor public benefit, and require disclosure of purpose, duration, and expected outcome.
- Technical Requirements and Governing Regulations: Access should be granted by ethics boards, institutional review boards, and PSOs to support academic missions and improve clinical care.

Stakeholder-specific data use has emerged as a critical aspect of surgical video data utilization. Various stakeholders, including patients, surgeons, researchers, scientific societies, healthcare institutions, MedTech companies, health insurances, and public institutions (e.g., regulatory institutions), use surgical video data for different purposes. The expert panel strongly agrees that these stakeholders use the data for a range of reasons, such as education, documentation, research, advertising, quality improvement, legal evidence, product development and policy development. Given the diverse interests of stakeholders, it is crucial to ensure that data access is use-case specific, consistently favors public benefit, and mandates disclosure of purpose, duration, and expected outcome taking ethical and legal aspects into account. This approach allows for better management of data usage, safeguarding the interests of all parties involved. Technical requirements and governing regulations should be designed to accommodate the various stakeholders and their use cases. Access to surgical video data should be granted by ethics and institutional review boards, as well as patient safety organizations (PSOs). This process ensures that the data usage aligns with the overall academic mission of improving clinical care while maintaining ethical standards. Moreover, the expert panel concurs that surgical video data collected by one stakeholder for a specific use may be accessed by multiple stakeholders for other uses, given that proper agreements and permissions are in place. This approach fosters collaboration, knowledge sharing, and innovation while maintaining the necessary privacy and ethical standards.

In conclusion, stakeholder-specific data use is essential for maximizing the benefits of surgical video data. By implementing appropriate technical requirements, governing regulations, and ethical guidelines, data access can be tailored to the needs of each stakeholder, ultimately promoting public benefit and improved clinical care.

### Next steps

Considering the insights gained from examining data use, data structure, data exploration, and data governance, several pivotal initiatives have been identified to propel surgical data science and optimize the utilization of surgical video data in AI applications:

- Establish Universal Guidelines: Collaborate with medical societies, researchers, and institutions to create standardized guidelines governing data collection, structuring, storage, and processing, thereby ensuring uniformity, compatibility, and dependability across various datasets resulting in a clear, pragmatic policy framework.
- Strengthen Data Privacy and Traceability: Develop technical solutions and legal frameworks that balance data traceability with patient privacy. This entails examining methods for deidentification and anonymization while preserving the capacity to reidentify data when necessary in compliance with applicable privacy regulations.
- Address Bias and Enhance Data Diversity: Foster research that tackles biases in surgical video data and advocates for data diversity. This may include formulating guidelines for dataset composition and incorporating metadata to help identify and mitigate biases within datasets.
- Encourage Interdisciplinary Cooperation: Facilitate collaboration among researchers, clinicians, institutions, and regulatory bodies to align technical advancements with clinical requirements and regulatory guidelines. This interdisciplinary dialogue will serve to bridge gaps between technology, clinical practice, and data governance.
- Improve Patient Education and Empowerment: Bolster patient education and awareness regarding their surgical video data usage and implications. Empowering patients to make well-informed decisions may minimize the need for subsequent consent withdrawal, thereby streamlining data acquisition processes.
- Advocate Ethical Data Usage: Ensure that access to surgical video data is determined by specific use cases, consistently promoting public benefit and monitored by ethics and institutional review boards as well as patient safety organizations (PSO). This approach will support an overarching academic mission aimed at enhancing clinical care.
- Foster Emerging Technologies and Research: Promote the development and exploration of innovative AI applications in surgical video data analysis to uncover new insights and improve patient outcomes. This includes backing open research studies and alleviating entry barriers for AI applications concerning data privacy, coverage, and alignment of novel technical capabilities with clinical needs.

## Conclusion

Comprehending the multifactorial aspects surrounding surgical video data and its associated metadata is crucial for harnessing its full potential in improving patient care, surgical practice, and medical research. This manuscript offers valuable insights into the perspectives of clinical, academic, and industrial experts in the field and presents select recommendations for managing data throughout its lifecycle. While the statements provided are not exhaustive or universally applicable, they represent a substantial attempt to establish guidelines for best practices in surgical data science. However, it is essential to recognize that more effort is needed to address the four key themes highlighted in this paper: data use, data structure, data exploration, and data governance. To ensure a comprehensive approach, it is vital to include currently underrepresented stakeholders, such as patient representatives and lawmakers, in future discussions and decision-making processes. Their involvement will help create a more inclusive and robust framework for surgical data science, ultimately leading to advancements in clinical care and surgical practice. By fostering a collaborative environment that involves diverse stakeholders and embraces a patient-centric approach, the surgical data science community can overcome current challenges and pave the way for groundbreaking innovations in AI applications, surgical video data analysis, and improved patient outcomes. This collective effort will help shape the future of surgery and patient care for years to come.

With the vast knowledge gained in this project, the crucial take-home messages and consensus recommendations are as follows:

- **Data use** Advocating for holistic data acquisition encourages the creation of larger and more diverse datasets. Methods of storing, processing, and sharing surgical video data must account for current and future use cases while adhering to ethical and legal regulations. This necessitates greater transparency of legal frameworks and practical, centrally formulated guidelines for stakeholders involved in creating and maintaining surgical video data.
- **Data structure** Conscious and informed decisions about data structure should be made at the individual institution level, as strict mandates could disincentivize recording efforts. These decisions should consider the trade-offs between:
  o Practicality and feasibility (storage capacity, cost, etc.),
  o Completeness of the data (recording all procedures, metadata, and other data modalities), and
  o Ideal data architecture (format, resolution, frame rate). Linking surgical video data with rich metadata and other data sources enhances compliance with data regulations and expands potential data use in research, education, and clinical practice.
  o Bias within datasets pertains to the innate structure of the data and associated metadata. While future technology may rely less on large amounts of perfectly annotated data, validation of algorithmic performance continues to require well-curated data. Therefore bias present a key topic to be addressed, monitored and moderated in all stages of the data lifecycle.
- **Data exploration** Datasets should be meticulously examined for innate biases, and research outputs should be required to discuss potential biases within the underlying data. Acquisition and synchronization of metadata alongside surgical video data offer more insight into potential biases. Data attributes should consider both existing applications of the data and future-proofing for short- and long-term emerging technologies.
- **Data governance** Greater insight into who is accessing the data, for what purpose, and when is essential. Regulating data privacy, consent, and differential access necessitates understanding the origin and ownership of the data, which relies on insights into currently underregulated and scarcely available metadata.

## References

[1] Gibaud B (2018). Toward a standard ontology of surgical process models. Int J Comput Assist Radiol Surg.

[2] Meireles OR (2021). SAGES consensus recommendations on an annotation framework for surgical video. Surg Endosc.

[3] Filicori F (2023). SAGES video acquisition framework-analysis of available OR recording technologies by the SAGES AI task force. Surg Endosc.

[4] Maier-Hein L (2022). Surgical data science—from concepts toward clinical translation. Med Image Anal.

[5] Kennedy-Metz LR (2021). Computer vision in the operating room: opportunities and caveats. IEEE Trans Med Robot Bionics.

[6] Kitaguchi D (2022). Artificial intelligence for computer vision in surgery: a call for developing reporting guidelines. Ann Surg.

[7] Yang JH (2022). Using AI and computer vision to analyze technical proficiency in robotic surgery. Surg Endosc.

[8] Bar O (2020). Impact of data on generalization of AI for surgical intelligence applications. Sci Rep.

[9] Pfahl A (2022). Combined indocyanine green and quantitative perfusion assessment with hyperspectral imaging during colorectal resections. Biomed Opt Express.

[10] Dennler C (2021). Augmented reality in the operating room: a clinical feasibility study. BMC Musculoskelet Disord.

[11] Ward TM (2021). Automated operative phase identification in peroral endoscopic myotomy. Surg Endosc.

[12] Bhatti KM (2021). Diagnostic performance of artificial intelligence-based models for the detection of early esophageal cancers in Barret’s esophagus: a meta-analysis of patient-based studies. Cureus.

[13] Maier-Hein L (2014). Crowdsourcing for reference correspondence generation in endoscopic images. Medical image computing and computer-assisted intervention—MICCAI 2014.

[14] Fjeld J, Achten N, Hilligoss H, Nagy A, Srikumar M (2020). Principled artificial intelligence: mapping consensus in ethical and rights-based approaches to principles for AI. SSRN Electron J.

[15] Ho D-A, Beyan O (2020) Biases in data science lifecycle. arXiv:2009.09795

[16] Kamath G et al (2023) A bias-variance-privacy trilemma for statistical estimation. arXiv:2301.13334

[17] Balestriero R et al (2023) A cookbook of self-supervised learning. arXiv:2304.12210

[18] Ouyang L (2022). Training language models to follow instructions with human feedback. Adv Neural Inf Process Syst.

[19] Radford A (2021). Learning transferable visual models from natural language supervision. International conference on machine learning.

[20] Brown TB (2020). Language models are few-shot learners. Adv Neural Inf Process Syst.

[21] Moor M (2023). Foundation models for generalist medical artificial intelligence. Nature.

[22] Bommasani R et al (2021) On the opportunities and risks of foundation models. arXiv:2108.07258

